# Real-Time Monitoring of the Effect of Tumour-Treating Fields on Cell Division Using Live-Cell Imaging

**DOI:** 10.3390/cells11172712

**Published:** 2022-08-31

**Authors:** Hoa T. Le, Michael Staelens, Davide Lazzari, Gordon Chan, Jack A. Tuszyński

**Affiliations:** 1 Department of Medical Microbiology & Immunology, Faculty of Medicine & Dentistry, University of Alberta, Edmonton, AB T6G 2E1, Canada; 2 Department of Physics, Faculty of Science, University of Alberta, Edmonton, AB T6G 2E1, Canada; 3 Dipartimento di Ingegneria Meccanica e Aerospaziale (DIMEAS), Politecnico di Torino, 10129 Turin, Italy; 4 Department of Oncology, Faculty of Medicine & Dentistry, University of Alberta, Edmonton, AB T6G 1Z2, Canada

**Keywords:** oncology, electromagnetic fields, non-invasive therapies, human cervical carcinoma cells, human breast epithelial cells, thymidine block, spinning disk microscopy, time-lapse microscopy

## Abstract

The effects of electric fields (EFs) on various cell types have been thoroughly studied, and exhibit a well-known regulatory effect on cell processes, implicating their usage in several medical applications. While the specific effect exerted on cells is highly parameter-dependent, the majority of past research has focused primarily on low-frequency alternating fields (<1 kHz) and high-frequency fields (in the order of MHz). However, in recent years, low-intensity (1–3 V/cm) alternating EFs with intermediate frequencies (100–500 kHz) have been of topical interest as clinical treatments for cancerous tumours through their disruption of cell division and the mitotic spindle, which can lead to cell death. These aptly named tumour-treating fields (TTFields) have been approved by the FDA as a treatment modality for several cancers, such as malignant pleural mesothelioma and glioblastoma multiforme, demonstrating remarkable efficacy and a high safety profile. In this work, we report the results of in vitro experiments with HeLa and MCF-10A cells exposed to TTFields for 18 h, imaged in real time using live-cell imaging. Both studied cell lines were exposed to 100 kHz TTFields with a 1-1 duty cycle, which resulted in significant mitotic and cytokinetic arrest. In the experiments with HeLa cells, the effects of the TTFields’ frequency (100 kHz vs. 200 kHz) and duty cycle (1-1 vs. 1-0) were also investigated. Notably, the anti-mitotic effect was stronger in the HeLa cells treated with 100 kHz TTFields. Additionally, it was found that single and two-directional TTFields (oriented orthogonally) exhibit a similar inhibitory effect on HeLa cell division. These results provide real-time evidence of the profound ability of TTFields to hinder the process of cell division by significantly delaying both the mitosis and cytokinesis phases of the cell cycle.

## 1. Introduction

Cancer is a ubiquitous disease among mammals; as the next-to-leading cause of death globally, a tremendous amount of research has been devoted to the development of successful treatment modalities. In most cases, primary cancer treatments begin with surgery, typically supplemented with additional treatments such as chemotherapy or radiotherapy afterwards. Although this approach can be highly effective at essentially eliminating many forms of cancer, it also comes with an overwhelming number of adverse effects. A promising non-invasive approach to treating various forms of cancer, with minimal side effects, involves the usage of electric fields (EFs). The regulatory effects of EFs on a variety of cell functions have been well studied [1,2]; for example, high-frequency EFs (MHz range) have been shown to cause the rapid oscillation of polar molecules, which leads to heating (through friction) [3]. Consequently, high-frequency EFs have been used to induce thermal tumour ablation [4]. At the other end of the spectrum, low-frequency alternating EFs (<1 kHz) have demonstrated an effect on cell membrane polarization [5]. Recently, intermediate-frequency alternating EFs (in the order of hundreds of kHz)—which historically have not been considered for medical applications [6]—have attracted significant interest as a treatment in oncology due to their remarkable ability to disrupt cancer cell division, discovered in the early 2000s [7]. A relatively new treatment modality for cancer has emerged as a result, referred to as tumour-treating fields (TTFields).

TTFields therapy uses low-intensity (1–3 V/cm) and intermediate-frequency (100–500 kHz) alternating EFs to treat various forms of cancer. TTFields exhibit an inhibitory effect on the proliferation of various cancer cell lines [7,8], indicating anti-mitotic activity. Two principal mechanisms of action have been proposed: First, TTFields cause dipole alignment, thereby inhibiting the polymerization of highly polarized tubulin subunits, resulting in the disruption of the mitotic spindle formation at metaphase. This leads to metaphase arrest, prolonged mitosis, abnormal chromosome segregation, and the formation of multinucleated cells, eventually leading to cell death [7,8,9]. Second, TTFields cause dielectrophoretic movement of polarized molecules to the region of high field density during cytokinesis, thereby concentrating large molecules and organelles to the mitotic furrow, disrupting cellular structures and causing cell fragmentation [7,8,10,11]. Previous studies have reported several factors that affect the efficacy of TTFields, including frequency, with cell-type-dependent optimal frequencies leading to the greatest reduction in cell counts [7,8,9,11,12,13]; electric field strength, with higher intensities causing an enhanced effect [7,8,13,14]; treatment duration, with longer treatments providing better results [9]; and the number of TTFields directions, with two-directional TTFields applied orthogonally favoured over a unidirectional field [8]. Additionally, there is a directionality dependence such that TTFields are more effective when their direction is parallel to the cell division axis [7,11,15].

Several additional lines of evidence indicate the anti-mitotic effects of TTFields and their subsequent benefits. TTFields treatment enhances cell membrane permeability [16,17], suggesting clinical implications in which co-treatment with TTFields may increase the uptake of therapeutic agents. Additionally, exposure to TTFields disrupts DNA damage repair capacity and induces replication stress [18,19]; this benefits therapies in which DNA-damage-inducing radiation or chemotherapy agents are employed. Recently, it has been demonstrated that TTFields induce changes in the organization and dynamics of microtubules and actin, thereby interfering with the cellular processes critical for cell adhesion and migration [9,20]; this is consistent with previous observations that TTFields exert anti-metastatic activity [21,22,23].

Glioblastoma multiforme (GBM)—the most common and malignant primary brain tumour, with an annual incidence of 3.19 per 100,000—has a mortality rate of approximately 75% in the first two years following diagnosis, and up to 90–95% upon reaching five years [24,25]. In 2011, TTFields received FDA approval as a monotherapy for recurrent GBM, with better results than chemotherapy in terms of efficacy, patient quality of life, and overall safety profile obtained in the randomized phase III clinical trial, EF-11 [6,26,27]. Subsequent FDA approval was granted in 2015 for combination therapy for newly diagnosed GBM (with adjuvant temozolomide therapy—a chemotherapy agent often used to treat brain tumours) based on the successful results of the phase III EF-14 clinical trial [24,28,29]. Several years later, in 2019, TTFields became FDA-approved for malignant pleural mesothelioma based on the results of the phase II STELLAR trial [30,31,32]—the first such treatment to receive FDA approval in over 15 years [33]. Additionally, several phase III randomized clinical trials investigating TTFields as a treatment for numerous other cancers are underway, including LUNAR (NCT02973789), for non-small-cell lung cancer [34]; EF-25/METIS (NCT02831959), for metastatic brain cancer [35,36]; ENGOT-ov50/GOG-3029/INNOVATE-3 (NCT03940196), for ovarian cancer [37,38]; and PANOVA-3 (NCT03377491), for pancreatic cancer [39]. For a relatively recent detailed review of the completed and ongoing TTFields clinical trials, see Ref. [33].

Although the biological effects of TTFields have been extensively investigated, their mechanisms of action against cancer cells are still not fully understood. Previous studies mostly provide limited and end-point information on the effects of TTFields (with only a few studies performing live-cell imaging throughout the TTFields exposure [9,20,40,41]). In this study, we employed the Novocure inovitro™ live system (Novocure Ltd., Haifa, Israel) to study the effects of exposure to TTFields on the division of HeLa and MCF-10A cells (human cervical carcinoma and breast epithelial cell lines, respectively) in real time, thereby providing a complete picture of the events occurring during the treatment. We begin with a description of this device and the other materials and methods used throughout this study in Section 2. Our results on the anti-mitotic effects of TTFields on HeLa and MCF-10A cells are presented in Section 3, and discussed in the subsequent section. Concluding remarks are provided in Section 5.

## 2. Materials and Methods

### 2.1. The Inovitro™ Live System

**Figure 1:**
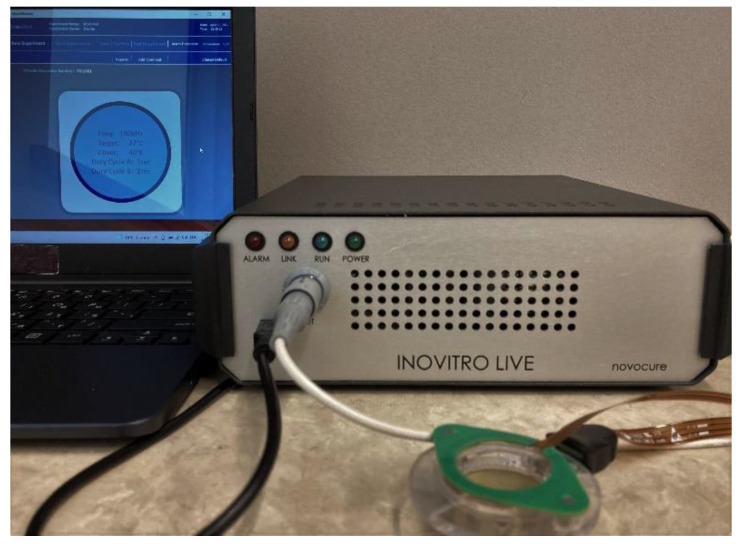
A digital photograph of the Novocure inovitro™ live system, with all four main components shown.

Several laboratory systems have been developed by Novocure Ltd. to facilitate studies of the effects of TTFields on cultured cells. In our studies, we used the Novocure inovitro™ live system, which allows one to monitor the effects of TTFields on cells in vitro in real time using live-cell imaging [42]. This system has been used in several past in vitro studies on the effects of TTFields on cancer cells (e.g., Refs. [20,40]). The inovitro™ live system comprises four main components: a TTFields generator, a cylindrical ceramic insert with a high dielectric constant that contains two orthogonal pairs of printed electrodes, a temperature-controlled heating element that covers the insert, and a laptop computer that controls the system via the inovitro™ live software. A digital photograph of the system is provided in Figure 1. The first component—the TTFields generator—produces low-intensity (1–3 V/cm) alternating EFs with an intermediate frequency in the range of 50–500 kHz, selected by the user. The ceramic insert containing the electrodes between which the electric field is produced is connected to the generator with the included cable and placed inside a 35 mm cell culture dish. Specifically, we placed the ceramic insert inside a 35 mm high-walled µ-dish containing 3 mL of medium. The temperature-controlled heating element is placed on the top of the insert, where it attaches magnetically. Lastly, the accompanying software that operates the system allows the user to control various parameters, i.e., the TTFields frequency, target medium temperature, cover heating element temperature, and the duty cycle (1-0 or 1-1, such that the TTFields alternate orientations between both perpendicular directions every second). The temperature of the heating element should be set above that of the target medium temperature (i.e., above the optimal temperature of 37 °C for cultured cells) to prevent condensation from developing on the cover of the ceramic insert. Moreover, it is necessary to set the ambient temperature below the target medium temperature to allow the TTFields generator to maintain the targeted temperature throughout the application (due to the small amount of heating caused by TTFields); thus, a lower ambient temperature is required for higher intensities of TTFields [43]. In this study, we set the target medium temperature to 37 °C and the ambient temperature to 23 °C.

**Figure 2:**
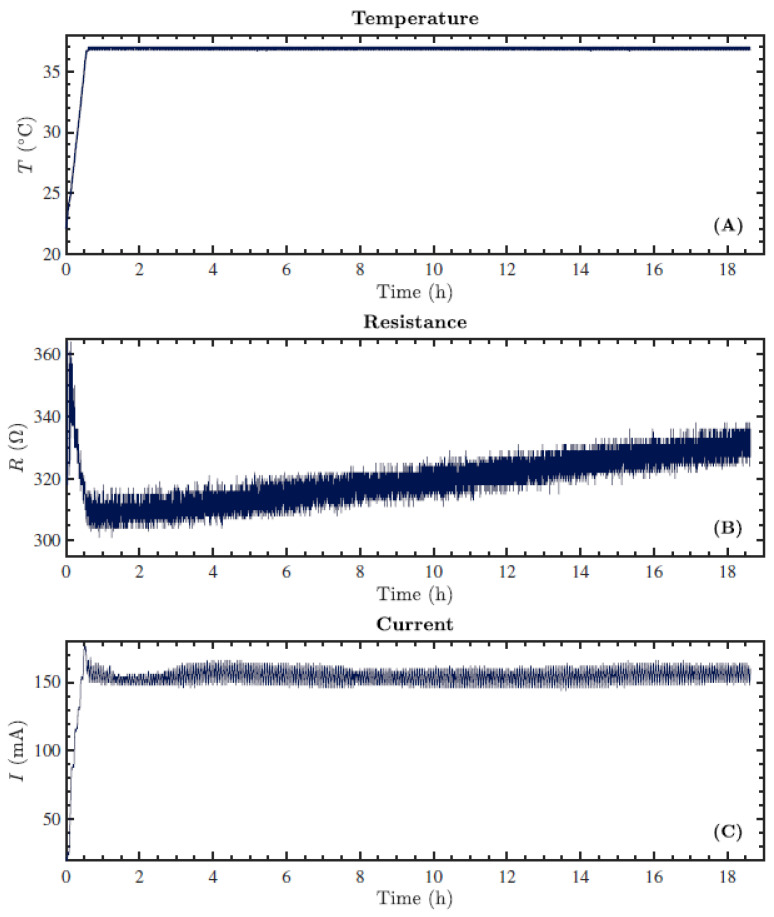
Representative graphs obtained from data logged every 3 s by the inovitro™ live system throughout the exposure of HeLa cells to TTFields with the following parameters: a frequency of 100 kHz, the target medium temperature set to 37 °C, a duty cycle of 1-1, and a duration of ~18 h. The ambient temperature was set to 23 °C. (**A**) The measured temperature (*T*) as a function of time. (**B**) The measured resistance (*R*) as a function of time. (**C**) The measured current (*I*) as a function of time.

Throughout the application of TTFields, the system also logs data on the medium temperature (*T*), resistance (*R*), and current (*I*) measured between both pairs of electrodes every 3 s, which are stored by the software. Figure 2 shows representative graphs of *T*, *R*, and *I* obtained after 18 h of treating HeLa cells with TTFields applied at a frequency of 100 kHz with a 1-1 duty cycle. The cells were maintained in a microscope box in an atmosphere of 5% CO_2_. It took approximately 40 min for the medium to reach 37 °C (Figure 2A). Afterwards, the current stabilized around 155 mA (Figure 2C), while the resistance slowly but constantly increased from approximately 310 to 330 Ω (Figure 2B).

### 2.2. Cell Culture

HeLa and MCF-10A cells stably expressing both H2B-mCherry and EGFP-tubulin (enhanced green fluorescent protein) labels were obtained at the University of Alberta’s Cross Cancer Institute. The HeLa cells were cultured in high-glucose Dulbecco’s Modified Eagle’s Medium (DMEM) supplemented with 5% (*v*/*v*) fetal bovine serum (FBS) and antibiotics (100 U/mL penicillin and 100 µg/mL streptomycin). The MCF-10A cells were cultured in DMEM/F12 supplemented with 20 ng/mL human epidermal growth factor (EGF), 1 mL of insulin, 500 ng/mL hydrocortisone, and 2 mM L-glutamine. Additionally, we added soybean trypsin inhibitor at a 1:1 molar ratio. Both cell types were cultured at 37 °C with 5% CO_2_ unless otherwise stated.

### 2.3. TTFields Treatment 

The unexposed control cells were maintained in a microscope chamber at a temperature of 37 °C in an atmosphere of 5% CO_2_. The cells receiving treatment with TTFields were placed in a 23 °C microscope chamber alongside the ceramic insert connected to the inovitro™ live system, and exposed to TTFields for 18 h. In this study, we employed the following settings: a target temperature of 37 °C, a cover temperature of 50 °C, a TTFields frequency of 100 or 200 kHz, and a duty cycle of 1-1 or 1-0.

### 2.4. Live-Cell Imaging

**Figure 3:**
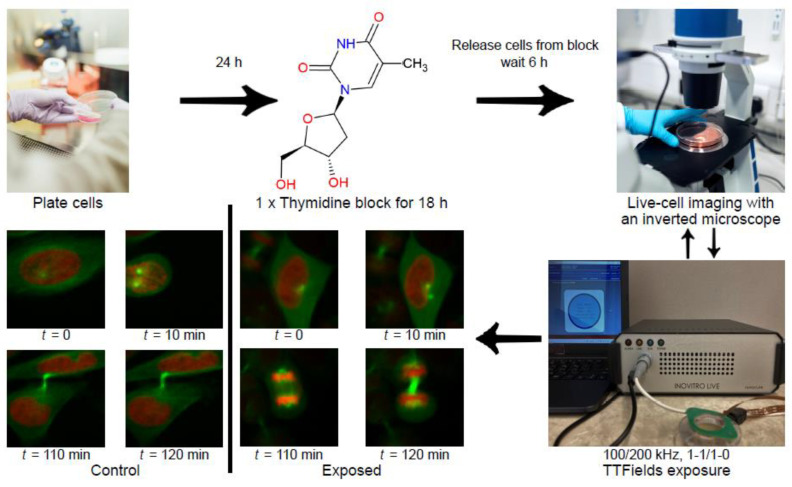
A schematic of the full procedure used in our live-cell imaging experiments with TTFields-exposed cells. The microscope images of exposed HeLa cells shown here were obtained in our experiments with 100 kHz TTFields with a 1-1 duty cycle.

HeLa or MCF-10A cells (3 × 10^4^ cells in a 0.5 mL drop) were seeded on the coverslip of a 35 mm ibidi high-walled glass-bottomed dish. The following day, the cells were synchronized in the G1/S phase by a single thymidine block. Briefly, the cells were treated with 2 mM thymidine, blocking the cell cycle for 18 h. Cells were released from the thymidine block for 6 h and then placed onto a microscope stage within an incubation chamber with an atmosphere of 5% CO_2_. Imaging was performed using a spinning disk (Ultraview, PerkinElmer, Waltham, MA, USA) on an inverted microscope (Axiovert 200 M; Carl Zeiss, Oberkochen, Germany) with a 40× objective lens. Notably, for the exposed cells, the TTFields were applied for 1 h before imaging to ensure that the target temperature reached 37 °C by the time of imaging. Z-stack images were acquired every 10 min for up to 17 h. The treated cells were not tracked beyond the 17 h duration of the live-cell imaging. A schematic of the full experimental procedure is provided in Figure 3.

### 2.5. Statistical Analysis

Merged z-stack images were analyzed using ImageJ v1.53q https://imagej.nih.gov/ij/ (accessed on 11 August 2022) [44,45] by tracking individual cells for mitotic and cytokinetic progression. We report the data from three independent experiments per group (the only exception is the MCF-10A control group, which includes data collected from two independent experiments) as the mean ± standard deviation (SD). In total, we measured the individual durations of mitosis for 164, 172, 205, and 195 HeLa cells in the control, 100 kHz (1-1), 100 kHz (1-0), and 200 kHz (1-1) TTFields scenarios, respectively. The same numbers of unexposed HeLa cells were analyzed for their cytokinesis durations, whereas in the TTFields-exposed samples, a fraction of the monitored cells had not completed cytokinesis over the 17 h imaging period; thus, in the experiments with 100 kHz (1-1), 100 kHz (1-0), and 200 kHz (1-1) TTFields, the cytokinesis durations were measured for only 162, 189, and 187 HeLa cells, respectively. In the experiments with MCF-10A cells, we measured the mitosis (cytokinesis) durations for 110 and 67 (108 and 56) cells in the control and TTFields-exposed groups, respectively. Two-sample independent *t*-tests were performed on both datasets (mitosis and cytokinesis durations) for both cell lines studied to establish whether the observed differences between the various groups of TTFields-exposed cells and the unexposed control cells were statistically significant. Additionally, for the experiments with HeLa cells, the datasets obtained for the different exposed groups studied were compared simultaneously using two-way analysis of variance (ANOVA) tests performed in MATLAB, MATLAB v9.11 (R2021b) Update 2 https://www.mathworks.com/products/matlab.html (accessed on 23 August 2022) to determine the levels of significance of the observed effects of independently varying the TTFields’ frequency and duty cycle. Additional information regarding these analyses and the complete results of each test are provided in the Supplementary Materials.

## 3. Results

### 3.1. TTFields Cause Mitotic and Cytokinetic Arrest in HeLa and MCF-10A Cells

The cell cycle consists of two major phases: interphase, which includes G1 (first gap), S (synthesis phase), and G2 (second gap); followed by the mitotic phase, consisting of mitosis and cytokinesis. During interphase, DNA is replicated, and cells grow to prepare for mitosis. The mitotic phase is subdivided into five phases—prophase, prometaphase, metaphase, anaphase, and telophase—based on the dynamic changes in chromosome morphology and movement. The mitotic spindle, formed from microtubules, orchestrates the movement of chromosomes. Cytokinesis is the final step in cell division, which starts at anaphase with the contraction of the plasma membrane to form a cleavage furrow, followed by the formation of the intercellular bridge composed of bundles of midbody microtubules aligned in an anti-parallel configuration, and finishing with the cleavage of the midbody to create two daughter cells—also called cytokinetic abscission. In this study, we used HeLa and MCF-10A cells expressing H2B-mCherry and EGFP-tubulin to monitor the dynamics of the chromosomes and microtubules during mitosis and cytokinesis. Individual cells were tracked by acquiring images of H2B-mCherry and EGFP-tubulin every 10 min to evaluate the time spent on both phases of this part of the cell cycle. After synchronization by thymidine, cells were released from the G1/S block for 6 h prior to imaging. Previous studies reported that it takes approximately 9–10 h for HeLa cells to begin mitosis after being released from the thymidine block [46,47]. Time-lapse images of the cells were acquired every 10 min for up to 17 h, either in the presence or absence of TTFields.

**Figure 4:**
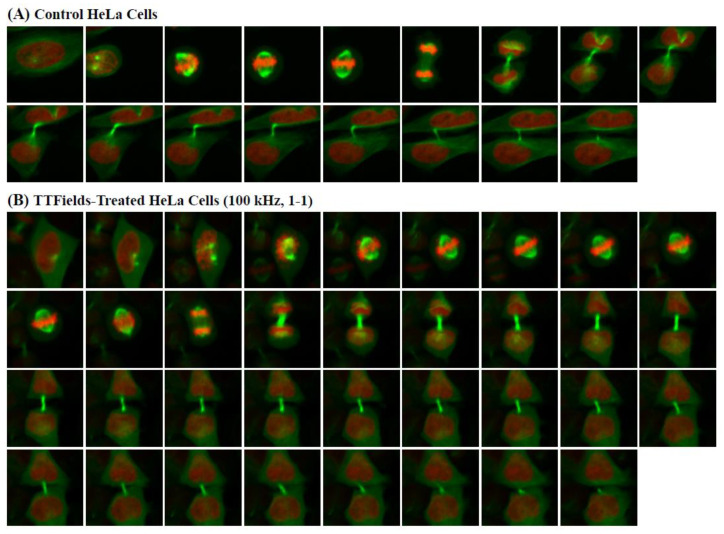
A total of 3 × 10^4^ HeLa cells were seeded on a coverslip of a 35 mm ibidi dish and allowed to adhere overnight. Cells were synchronized in the G1/S phase by a single thymidine block and then released for 6 h before imaging. Cells were maintained in an atmosphere of 5% CO_2_ with an ambient temperature of 37 °C for the control cells and 23 °C for the TTFields-treated cells. (**A**) Representative images of unexposed HeLa cells acquired every 10 min throughout cell division. (**B**) Representative images of TTFields-treated HeLa cells (100 kHz, 1-1 duty cycle) acquired every 10 min throughout cell division.

**Figure 5:**
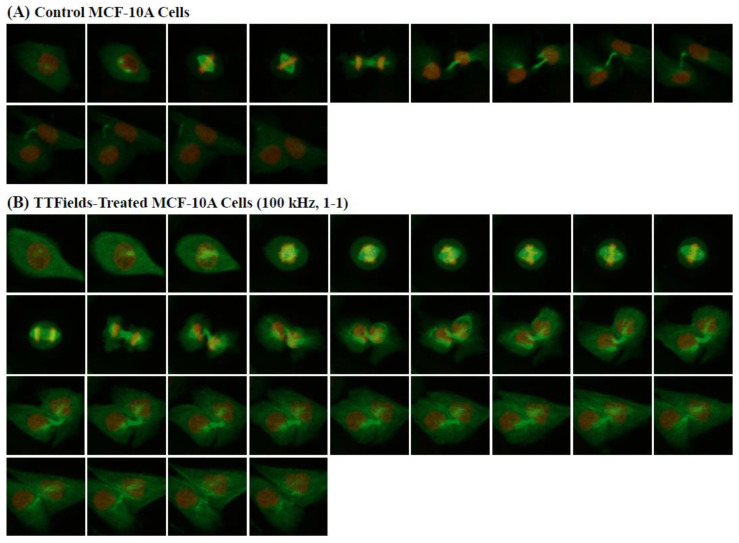
A total of 3 × 10^4^ MCF-10A cells were seeded on a coverslip of a 35 mm ibidi dish and allowed to adhere overnight. Cells were synchronized in the G1/S phase by a single thymidine block and then released for 6 h before imaging. Cells were maintained in an atmosphere of 5% CO_2_ with an ambient temperature of 37 °C for the control cells and 23 °C for the TTFields-treated cells. (**A**) Representative images of unexposed MCF-10A cells acquired every 10 min throughout cell division. (**B**) Representative images of TTFields-treated MCF-10A cells (100 kHz, 1-1 duty cycle) acquired every 10 min throughout cell division.

As previously mentioned, the component of the inovitro™ live system that is in contact with the cells is the ceramic insert. Thus, to ensure that the ceramic insert would not affect the results, the insert was placed in both sham control and TTFields-treated groups. Initially, the TTFields were applied with the following settings: a frequency of 100 kHz, a duty cycle of 1-1, the target temperature set to 37 °C, and an ambient temperature of 23 °C. Representative images of the control and TTFields-treated cells are shown in Figure 4A,B for the HeLa cells, respectively, and Figure 5A,B for the MCF-10A cells, respectively.

**Figure 6:**
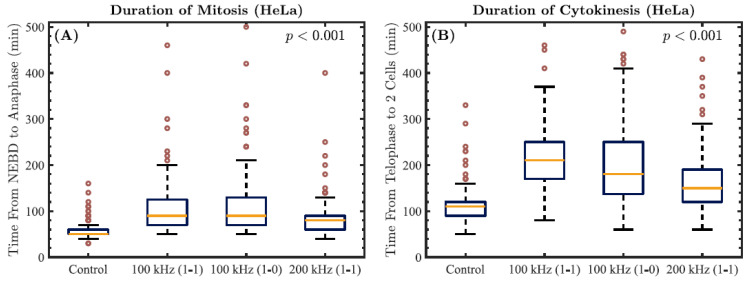
The durations of mitosis and cytokinesis obtained from three independent experiments with HeLa cells in a variety of scenarios: control, 100 kHz (1-1 duty cycle), 100 kHz (1-0), and 200 kHz (1-1). The *p*-value overlaid on each plot corresponds to the results obtained from two-sample *t*-tests comparing the displayed data for HeLa cells in each TTFields-exposed group to those of the control cells. (**A**) Boxplots representing the duration of mitosis, calculated from nuclear envelope breakdown (NEBD) to anaphase. (**B**) Boxplots representing the duration of cytokinesis, calculated from telophase to the formation of two separated daughter cells. Data are displayed as the median duration flanked by upper and lower quartiles, with outliers shown as red circles.

In this study, we analyzed more than 160 HeLa cells from three independent experiments (per experimental group) for their durations of mitosis and cytokinesis. The former was measured as the time (in minutes) from nuclear envelope breakdown (NEBD) to anaphase (when the chromosomes move away from one another), while the latter was measured from telophase (when the chromosomes reach opposite poles and start to decondense) to the formation of two separated daughter cells. Only the cells that started mitosis during the first 10 h of imaging and had at least completed mitosis by 17 h of imaging were analyzed. Eighty percent of the HeLa cells in the control group completed the entire process within 3 h. In the TTFields-treated group, only 3.5% could complete both mitosis and cytokinesis within 3 h, indicating a profound effect of TTFields on HeLa cell division. The control HeLa cells had a median duration of mitosis of 50 min, while the cells treated with 100 kHz TTFields with a 1-1 duty cycle exhibited a significant increase in this time by 40 min (*p <* 0.001), as shown in Figure 6A. Similarly, for this choice of parameters, the TTFields-treated HeLa cells exhibited a significantly longer median cytokinesis duration than the control cells—210 min compared to 110 min, respectively (Figure 6B, *p <* 0.001). As a result of the exposure to TTFields with these parameters, both processes took roughly twice as long, demonstrating that both mitosis and cytokinesis in HeLa cells are severely affected by TTFields.

In the scenario involving HeLa cells treated with 100 kHz TTFields with a 1-0 duty cycle, the increase in the median duration of mitosis compared to the control group was equivalent to that obtained when we used a 1-1 duty cycle (Figure 6A, *p <* 0.001). Moreover, no statistically significant differences in the duration of mitosis were found when varying the duty cycle (*p >* 0.1). However, the induced cytokinetic arrest was found to be less substantial when a 1-0 duty cycle was used instead of a 1-1 duty cycle (Figure 6B, 0.01 *< p <* 0.05). When the TTFields frequency was increased to 200 kHz with a 1-1 duty cycle, we observed a significant overall reduction in the effects (*p <* 0.001), as both phases were less restrained (Figure 6).

**Figure 7:**
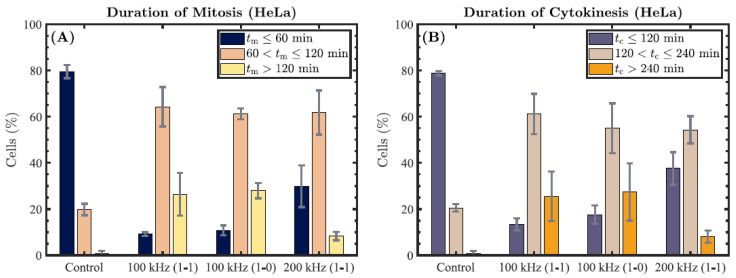
The HeLa cells from each set of experiments were grouped based on the length of mitosis (*t*_m_ ≤ 60 min, 60 < *t*_m_ ≤ 120 min, or *t*_m_ > 120 min) and the length of cytokinesis (*t*_c_ ≤ 120 min, 120 < *t*_c_ ≤ 240 min, or *t*_c_ > 240 min). Graphs represent the percentage of the cells in each category, reported as the mean ± SD from three independent experiments. (**A**) Results obtained by grouping the control and TTFields-exposed HeLa cells by their duration of mitosis. (**B**) Results obtained by grouping the control and TTFields-exposed HeLa cells by their duration of cytokinesis.

Furthermore, we divided the mitotic HeLa cells into three groups based on the duration of mitosis—*t*_m_ ≤ 60 min, 60 < *t*_m_ ≤ 120 min, and *t*_m_ > 120 min—for each of the previously stated choices of TTFields parameters studied (Figure 7A). We calculated the percentage of cells in each group. The results are presented as the mean (±SD) obtained from the three independent experiments. Over 50 HeLa cells were analyzed per experiment. In the control group, 79.5 ± 2.8% of the cells had a mitosis duration of *t*_m_ ≤ 60 min, and 19.8 ± 2.5% had a duration of 60 < *t*_m_ ≤ 120 min. In the 100 kHz TTFields-treated group (1-1 duty cycle), 64.3 ± 8.5% had a mitosis duration of 60 < *t*_m_ ≤ 120 min, and 26.4 ± 9.3% had a duration of over 120 min. Similarly, based on the length of cytokinesis, the cells were divided into another three groups: *t*_c_ ≤ 120 min, 120 < *t*_c_ ≤ 240 min, and *t*_c_ > 240 min (Figure 7B). In the control group, 78.8 ± 1.0% of the cells had a cytokinesis duration of *t*_c_ ≤ 120 min, and 20.5 ± 1.6% had a duration of 120 < *t*_c_ ≤ 240 min. In the TTFields-treated group (100 kHz, 1-1), 61.1 ± 8.8% had a cytokinesis duration of 120 < *t*_c_ ≤ 240 min, and 25.5 ± 10.8% had a duration of over 240 min. These results suggest that subpopulations of HeLa cells are more sensitive to TTFields exposure (likely due to the respective alignments of their axes of cell division with respect to the orientation of the TTFields).

**Figure 8:**
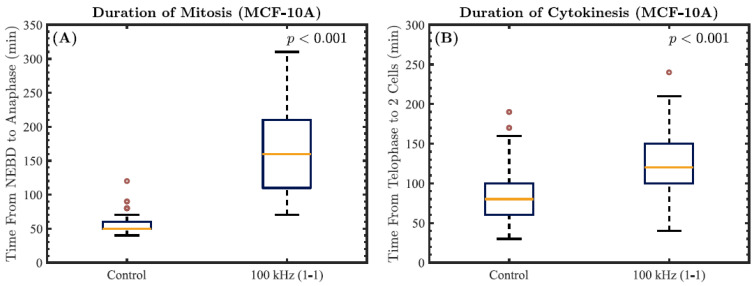
The durations of mitosis and cytokinesis obtained from our experiments with MCF-10A cells. The *p*-value overlaid on each plot corresponds to the results obtained from each two-sample *t*-test comparing the displayed data for MCF-10A cells in the TTFields-exposed group to those of the control cells. (**A**) Boxplots representing the duration of mitosis, calculated from nuclear envelope breakdown (NEBD) to anaphase. (**B**) Boxplots representing the duration of cytokinesis, calculated from telophase to the formation of two separated daughter cells. Data are displayed as the median duration flanked by upper and lower quartiles, with outliers shown as red circles.

In our experiments with MCF-10A cells, more than 50 cells were analyzed for their mitotic and cytokinetic durations in both the control and TTFields-exposed groups. As was observed in our experiments with HeLa cells, exposure to 100 kHz TTFields with a 1-1 duty cycle also caused mitotic and cytokinetic arrest in MCF-10A cells, as shown in Figure 8A,B, respectively. Specifically, the median duration of mitosis for the TTFields-exposed MCF-10A cells was found to be 110 min longer than that for the control group. In the case of the median cytokinesis durations obtained, a smaller increase of 40 min was found in the TTFields-exposed group. Based on the results of the two-sample *t*-tests applied in our statistical analysis of this dataset, both of these outcomes were found to be highly statistically significant (*p <* 0.001).

Finally, several videos following a few individual control and TTFields-exposed HeLa cells throughout cell division were created from the live-cell images using ImageJ. Real-time tracking of the dynamics of chromosomes (in red) and microtubules (in green) in the mitotic cells was enabled through their labelling with H2B-mCherry and EGFP-tubulin, respectively. These videos are provided in the Supplementary Materials as Videos S1–S5, alongside a supplementary document that includes additional information regarding their creation. The first two videos (Videos S1 and S2) follow two individual unexposed HeLa cells as they undergo mitosis and cytokinesis. These processes are unhindered and occur relatively quickly, with the initial alignment and segregation of chromosomes and the formation of spindle fibers easily visible via their respective markers. This is followed by the separation of the cell into two daughter cells connected by an intercellular bridge, which is disconnected shortly thereafter through cytokinetic abscission. In the case of HeLa cells exposed to 100 kHz TTFields with a 1-0 duty cycle, these processes are strongly inhibited, as shown in Videos S3–S5. In particular, Video S3 provides a good example of improper chromosome alignment and segregation resulting from TTFields exposure, leading to multinucleated daughter cells (an indicator of mitotic catastrophe [9,48]). In all three of these supplementary videos, the TTFields undoubtedly induce a restraining effect on the mitotic cells, as the entire process is noticeably delayed. Moreover, the intercellular bridge between daughter cells appears to remain connected, thereby indicating a significant disruption in the behaviour of microtubules.

### 3.2. Single and Two-Directional TTFields Exhibit Similar Inhibitory Effects on HeLa Cell Division

As mentioned earlier, to cover multiple orientation axes of cell division, the ceramic insert contains two perpendicular pairs of electrodes that switch the TTFields between both directions at the frequency set by the duty cycle. Previous reports suggest that two-directional TTFields applied orthogonally are more effective than unidirectional fields [8]. Therefore, we investigated whether turning off one pair of electrodes altered the efficacy of TTFields in prolonging the duration of mitosis and cytokinesis in HeLa cells. However, we did not detect any significant differences in the mitosis duration (*p* > 0.1) when unidirectional TTFields were applied compared to two-directional TTFields (Figure 6A and Figure 7A; 100 kHz, 1-1 vs. 100 kHz, 1-0). In the case of cytokinesis, the median duration obtained for cells exposed to two-directional TTFields (100 kHz, 1-1 duty cycle) was 30 min longer than when only one direction was used (Figure 6B). However, the statistical significance of this result was low (0.01 < *p* < 0.05), suggesting only weak evidence of such an effect. Hence, regarding the overall inhibition of the mitotic phase in HeLa cells, our results suggest that only a small benefit (if any) is obtained by employing orthogonally oriented two-directional TTFields. Furthermore, these lines of evidence indicate that applying 100 kHz TTFields along a single direction is sufficient to cause mitotic and cytokinetic arrest in HeLa cells.

### 3.3. The Anti-Mitotic Effect of TTFields on HeLa Cells Is Stronger at 100 kHz vs. 200 kHz

Previous studies reported that optimal frequencies lead to the highest reductions in cell counts [7,8,9,11,12,13]. Giladi et al. employed a frequency of 150 kHz to study the effects of TTFields on HeLa cells, and found that treatment with TTFields caused mitotic cell death and death at metaphase [9]. However, the authors did not provide data on any other frequencies. In this study, we compared the effects of TTFields applied at 100 and 200 kHz (with a 1-1 duty cycle) on the duration of mitosis and cytokinesis in HeLa cells. We found that TTFields applied at 200 kHz were demonstrably less effective, as their inhibitory effect on mitosis and cytokinesis was significantly weaker (*p* < 0.001) than that observed at 100 kHz (Figure 6 and Figure 7; 100 kHz, 1-1 vs. 200 kHz, 1-1).

## 4. Discussion

The efficacy of TTFields as a treatment for different types of cancer is known to depend greatly on the EF frequency, intensity, treatment duration, and the number of fields used, with perpendicular two-directional EFs being optimal over unidirectional fields. Our results presented in this study provide additional data on the frequency-dependence of the effects of TTFields on cultured HeLa cells in vitro, and on the potential benefits gained from employing two-directional versus unidirectional TTFields. Here, we contrast our results with previous studies of TTFields-exposed HeLa cells. The optimal effective TTFields frequency for treating various cancers appears to be cell-type-dependent [8,12]. For example, past research in both the laboratory and clinical settings has established that 200 kHz TTFields are the most effective at hindering the proliferation of ovarian cancer cells [13,43] and glioma cells [7,8,9,43,49], and inducing cell death. In the case of mesothelioma, 150 kHz TTFields have been established as the optimal frequency [50]. Limited studies exist on the frequency-dependent effects of TTFields on HeLa cells, with the majority of studies providing results for a single effective frequency in the range of 100–200 kHz [9,51], or for two different frequencies (e.g., 150 vs. 500 kHz, with the former demonstrating a stronger effect [11]). Moreover, there is some contention in the current results regarding the optimal effective frequency for the treatment of HeLa cells. Giladi et al. stated an optimal effective TTFields frequency of 150 kHz [9], which was subsequently reported in Ref. [52]. On the other hand, in the experiments by Li et al., who studied a range of TTFields frequencies from 100–1000 kHz, the inhibition ratio of HeLa cell growth decreased as the frequency was increased, with the maximal growth inhibition observed for 100 kHz TTFields [53]. Our results are consistent with their findings that, for the HeLa cell line, lower frequencies of TTFields are more effective at reducing cell counts and hindering cell division—a result that is likely, at least in part, due to cell morphology [54].

Indeed, it has also been demonstrated that the optimal effective frequency is cell-size-dependent [7,8]. In particular, this dependence appears to be such that lower-frequency TTFields (~100 kHz) are more effective against cells of larger size [8,55]. The ranges of cell diameters reported for glioma and mesothelial cells are ~5–20 µm [56,57] and ~10–50 µm [58], respectively. The sizes of mesothelial cells and HeLa cells are of the same order, with HeLa cells having a median surface area of ~550 µm^2^ [59] (values of 1600 ± 500 µm^2^ and 370 ± 100 µm^2^ are also reported in the literature for cells grown in pooled human serum and non-human serum, respectively [60]), corresponding to a range of diameters of ~20–40 µm [61,62]. However, compared to glioma cells, HeLa cells are roughly twice as large. Hence, our results on HeLa cells appear consistent with the claim that the optimal TTFields frequency is lower for larger cells. Additionally, findings indicate that cells with shorter doubling times are significantly more affected by TTFields [63]. This suggests that HeLa cells, which have a doubling time of ~33–35 h [64]—relatively similar to that of glioma (~23 h to a few days [65,66,67]) and mesothelioma (~30–72 h [68]) cells—should also be seriously affected by TTFields. This was confirmed by the significant mitotic and cytokinetic arrest observed in our experiments with TTFields-exposed HeLa cells.

As previously stated, many studies have also reported that two-directional TTFields applied perpendicularly are optimal over unidirectional fields, with improvements as large as ~20% observed [8,55] (in terms of the reduction in the cell count and the treated tumour volume). Regarding the effects of two-directional TTFields on the duration of the mitotic phase of the cell cycle, we found weak evidence (0.01 < *p* < 0.05) that for HeLa cells, only the duration of cytokinesis was prolonged to a greater extent when a 1-1 duty cycle was used compared to a 1-0 duty cycle; the duration of mitosis was seemingly unaffected, and any slight differences in the mean mitotic durations obtained for both experimental groups lacked statistical significance (*p* > 0.1). Thus, in the context of mitotic and cytokinetic arrest induced in TTFields-exposed HeLa cells, we did not find any highly significant differences in the efficacy of TTFields applied along two orthogonal directions compared to only one. Nevertheless, this may vary for different cell types.

In our experiments with the MCF-10A cell line—which is representative of normal human breast epithelial cells, and is frequently used as an in vitro model to study the behaviour of normal breast cells—we found that exposure to TTFields (100 kHz, 1-1) resulted in significant mitotic and cytokinetic arrest (*p* < 0.001). However, we did not observe a prevalence of multinucleated progeny, as was seen with the TTFields-exposed HeLa cells. As far as we are aware, this is the only study to date that has investigated the effects of TTFields on MCF-10A cells. Although there have been many successful clinical trials with TTFields, and their in vivo safety has been established in several studies [21,54], there are limited data available on the effects of TTFields on normal cells. In the recent work of Ye et al., it was found that TTFields applied to normal brain organoids in vitro induced cell damage that could lead to apoptosis [69]. In combination with our results, these findings strongly imply that treatment with TTFields should be accurately directed at tumour sites and applied such that the exposure of healthy tissues is minimized. Furthermore, these results suggest that more studies on the effects of TTFields on normal cells are highly warranted.

In the supplementary videos provided, we demonstrated that TTFields disrupt the behaviour of chromosomes and microtubules throughout cell division. These latter structures are highly polar in the mitotic phase of the cell cycle [70], suggesting that they could be vulnerable to the effects of external EFs. Indeed, several studies have demonstrated that TTFields have an effect on the dynamics and organization of microtubules (and actin filaments) [9,20]. In the work of Giladi et al., the structural properties of the mitotic spindle were analyzed by studying the MDA-MB-231 (breast adenocarcinoma) and A549 (lung adenocarcinoma) cell lines after exposure to 150 kHz TTFields for 24 h [9]. The authors discovered that exposure to TTFields reduced the percentage of polymerized microtubules and interfered with the structure of the mitotic spindle [9]. These effects were found to lead to abnormal segregation of chromosomes, multinucleated progeny and, ultimately, to cell death [9]. Supplementary Videos S3–S5 provide further confirmation of such effects. For example, noticeable effects on mitotic spindle microtubules are apparent in the TTFields-exposed HeLa cells compared to the control cells (Videos S1 and S2), providing important insight into the mode of action of TTFields. In particular, this real-time evidence directly supports the hypothesis that highly polar structures such as tubulin are greatly affected by TTFields, resulting in anomalous behaviour throughout mitosis and cytokinesis. Finally, although the mitotic cells in our study were not monitored beyond the 17 h period of live-cell imaging (and 18 h exposure to TTFields), such data are available in the literature. Specifically, the previously mentioned study by Giladi et al. found that upon observing the progeny of TTFields-exposed HeLa cells, either one or both daughter cells experienced cell death after the induced mitotic arrest (totalling 67% of the mitotic cells) within 21.5 ± 19.4 h after mitosis [9].

## 5. Conclusions

In the last decade, TTFields therapy has gained prominence as an effective cancer treatment modality with no major side effects. Many clinical trials and experimental studies of their remarkable inhibitory effects on different types of cancer cells have been conducted. Nevertheless, their mechanisms of action are still not entirely clear, and the current knowledge on cell-type-specific parameters that optimize their efficacy is incomplete. Accordingly, we sought to investigate the anti-mitotic effects of TTFields in real time by performing live-cell imaging of HeLa and MCF-10A cells expressing dual protein markers, enabling the monitoring of microtubule and chromosome dynamics simultaneously. We performed time-lapse microscopy throughout exposures of HeLa cells to TTFields with a frequency of either 100 or 200 kHz and a duty cycle of 1-1 or 1-0 to determine the individual durations of mitosis and cytokinesis of over 160 different mitotic cells per sample (from three independent experiments), and compared these durations with those obtained for unexposed control cells. In our studies, 100 kHz TTFields applied along two orthogonal directions exerted the maximal anti-mitotic effect on HeLa cells. However, there was only a marginal enhancement in the observed effects when compared to unidirectional TTFields applied at the same frequency, and this difference lacked strong statistical significance.

In our experiments with MCF-10A cells, which analyzed over 50 different mitotic cells per sample, exposure to 100 kHz TTFields with a 1-1 duty cycle also caused significant mitotic and cytokinetic arrest. Finally, we provided several videos comparing cell division in unexposed and TTFields-exposed HeLa cells. In the latter scenario, we observed a significant inhibition of HeLa cell division, indicated by an overall delay in the process, and characterized by abnormal microtubule and chromosome behaviour. These results complement the existing literature regarding the effects of TTFields on cancerous and non-cancerous cells in vitro, and further implicate their potential use as a treatment for human cervical cancers.

## Data Availability

The data presented in this study are openly available through FigShare at doi:10.6084/m9.figshare.19653993.

## Supplementary Materials

The following supporting information can be downloaded at: https://www.mdpi.com/article/10.3390/cells11172712/s1, Video S1: Control HeLa cells’ mitosis 1; Video S2: Control HeLa cells’ mitosis 2; Video S3: TTFields-exposed HeLa cells’ mitosis 1; Video S4: TTFields-exposed HeLa cells’ mitosis 2; Video S5: TTFields-exposed HeLa cells’ mitosis 3.
