# Supplementary material for: Real-Time Monitoring of the Effect of Tumour-Treating Fields on Cell Division Using Live-Cell Imaging
Source: Cells. 2022 Aug 31;11(17):2712. doi: 10.3390/cells11172712 (PMC9454843; doi:10.3390/cells11172712)
Supplement: Supplementary file 1 [file cells-11-02712-s001.zip › SuppMat.pdf]

Supplementary Materials: Real-Time Monitoring of the Effect of Tumour-Treating Fields on Cell Division Using Live-Cell Imaging

Hoa T. Le, Michael Staelens, Davide Lazzari, Gordon Chan and Jack A. Tuszyński

1. Statistical Tests

In order to establish the level of significance of the observed differences in the durations of cell division for HeLa cells exposed to TTFields, several statistical tests were performed between the various datasets obtained. The significance of our results obtained for the mitosis and cytokinesis durations of TTFields-exposed HeLa cells compared to the unexposed control cells was investigated using two-sample independent *t*-tests. This was followed by two-way analysis of variance (ANOVA) tests to simultaneously compare the datasets obtained for TTFields-exposed HeLa cells with differing choices of the two independent parameters studied, namely, the TTFields frequency and duty cycle. In the case of the MCF-10A cells, which had only one treatment group, we have exclusively applied two-sample independent *t*-tests in our statistical analysis.

1.1. Two-Sample Independent t-Test Results

The mitosis and cytokinesis durations measured for the control and TTFields-exposed HeLa and MCF-10A cells were compared using Welch's *t*-test, which assumes that these datasets consist of independent samples that are normally distributed with different variances. The latter two assumptions were validated by plotting histograms of each dataset and calculating their standard deviations. In Welch's *t*-test, the value of the *t* statistic is computed as follows,

$$t = \frac{\bar{x} - \bar{y}}{\frac{\sigma_x^2}{N_x} + \frac{\sigma_y^2}{N_y}}, \quad \text{(S1)}$$

where $\bar{x}$ and $\bar{y}$ represent the means of the two samples being compared, σ_x and σ_y represent their standard deviations, and N_x and N_y are the corresponding sample sizes. The two-sample *t*-tests were performed using MATLAB® with the `ttest2` function provided in the Statistics and Machine Learning Toolbox. A significance level of $\alpha = 0.05$ (default) was used. All of the parameters required to compute the corresponding *t*-values for the mitosis and cytokinesis datasets for both cell lines studied are provided in Tables S1 and S2, respectively. The complete results of each *t*-test performed are available in Tables S3 and S4, respectively. Notably, the results of all the *t*-tests performed, which compared the TTFields-exposed cells in each scenario against the unexposed control cells, are consistent with a rejection of the null hypothesis. Moreover, all of these results are highly statistically significant ($p < 0.001$).

Table S1. The statistics corresponding to the measured mitosis durations for the control and TTFields-exposed HeLa and MCF-10A cells: N represents the number of cells studied in each sample, $\bar{t}_m$ is the average duration of mitosis, and σ is the standard deviation.

Cell Type	Sample	N	$\bar{t}_m$	σ
HeLa	Control	164	58.41 min	18.89 min
	100 kHz (1-1)	172	106.28 min	54.73 min
	100 kHz (1-0)	205	112.49 min	63.24 min
	200 kHz (1-1)	195	84.62 min	37.06 min
MCF-10A	Control	110	54.64 min	12.39 min
	100 kHz (1-1)	67	164.93 min	63.35 min

Table S2. The statistics corresponding to the measured cytokinesis durations for the control and TTFields-exposed HeLa and MCF-10A cells: N represents the number of cells studied in each sample, $\bar{t}_c$ is the average duration of cytokinesis, and σ is the standard deviation.

Cell Type	Sample	N	$\bar{t}_c$	σ
HeLa	Control	164	113.11 min	38.75 min
	100 kHz (1-1)	162	215.93 min	74.37 min
	100 kHz (1-0)	189	198.36 min	84.11 min
	200 kHz (1-1)	187	160.11 min	61.01 min
MCF-10A	Control	108	83.43 min	33.72 min
	100 kHz (1-1)	56	123.04 min	37.02 min

Table S3. The results of various Welch's t-tests applied to the datasets containing the durations of mitosis for unexposed and TTFields-exposed HeLa and MCF-10A cells. Here, h_{null} represents the test decision regarding the rejection of the null hypothesis such that a value of 1 indicates a rejection of the null hypothesis at the 5% significance level (set by α).

Cell Type	Scenario	t-value	D.o.F	h_{null}	p-value	|Lower C.I.|	|Upper C.I.|
HeLa	Control vs. 100 kHz (1-1)	−10.81	212.93	1	5.25×10^{-22}	39.14 min	56.59 min
	Control vs. 100 kHz (1-0)	−11.61	248.20	1	3.49×10^{-25}	44.90 min	63.25 min
	Control vs. 200 kHz (1-1)	−8.63	298.51	1	3.75×10^{-16}	20.23 min	32.18 min
MCF-10A	Control vs. 100 kHz (1-1)	−14.09	69.09	1	5.40×10^{-22}	94.67 min	125.91 min

Table S4. The results of various Welch's t-tests applied to the datasets containing the durations of cytokinesis for unexposed and TTFields-exposed HeLa and MCF-10A cells. Here, h_{null} represents the test decision regarding the rejection of the null hypothesis such that a value of 1 indicates a rejection of the null hypothesis at the 5% significance level (set by α).

Cell Type	Scenario	t-value	D.o.F	h_{null}	p-value	|Lower C.I.|	|Upper C.I.|
HeLa	Control vs. 100 kHz (1-1)	−15.63	241.74	1	1.62×10^{-38}	89.86 min	115.78 min
	Control vs. 100 kHz (1-0)	−12.49	272.41	1	1.31×10^{-28}	71.81 min	98.69 min
	Control vs. 200 kHz (1-1)	−8.72	319.34	1	1.58×10^{-16}	36.39 min	57.60 min
MCF-10A	Control vs. 100 kHz (1-1)	−6.70	102.71	1	1.17×10^{-9}	27.88 min	51.34 min

1.2. Two-Way Analysis of Variance Results

We performed two-way ANOVA tests to simultaneously compare the means of the various (unbalanced) datasets obtained for the mitotic and cytokinetic durations of TTFields-exposed HeLa cells and to determine the statistical significance of our results for both factors tested: the TTFields frequency and the duty cycle. For two factors that vary independently and normally around a mean, y_{ab} (with constant variance), the model can be written as follows,

$$y_{ab} = \mu + \alpha_a + \beta_b + (\alpha\beta)_{ab} + \epsilon_{abr}, \tag{S2}$$

where y_{ab} is the mean of the response variable, $a \in \{1, .., i\}$ represents the levels of factor A with a total of i levels, and $b \in \{1, .., j\}$ represents the levels of factor B with a total of j levels. On the right hand side of Equation S2, μ represents the overall mean, α_a are the deviations of levels of factor A from μ due to A, β_b are the deviations of levels of factor B from μ due to B, ϵ_{abr} represents the statistical noise (assumed to be independent and normally distributed with constant variance), and $r \in \{1, ..., n_{ab}\}$ represents the replication number out of n_{ab} total replicates for a particular treatment (a, b). These tests were performed on the mitosis and cytokinesis duration datasets for TTFields-exposed HeLa cells using the `anovan` function in MATLAB®, which is also provided in the Statistics and Machine Learning Toolbox. The results are presented in Tables S5 and S6, respectively. Regarding the effects of the TTFields frequency (100 vs. 200 kHz) on the mitosis and cytokinesis durations of HeLa cells, the results obtained for both ANOVA tests indicated a rejection of the null hypothesis at a highly statistically significant level ($p < 0.001$). However, in the case of the effect of changing the duty cycle (1-1 vs. 1-0), only the observed difference in the cytokinesis duration was found to have any statistical significance, albeit weakly ($0.01 < p < 0.05$).

Table S5. The results of a two-way ANOVA test applied to the dataset containing the mitotic durations measured for HeLa cells exposed to TTFields with a frequency of 100 or 200 kHz and either a 1-1 or 1-0 duty cycle.

Factor	Sum. Sq.	D.o.F	Mean Sq.	F	Prob$>F$
Frequency	42890.6	1	42890.6	15.31	1.02×10^{-4}
Duty Cycle	3605.3	1	3605.3	1.29	0.257

Table S6. The results of a two-way ANOVA test applied to the dataset containing the cytokinetic durations measured for HeLa cells exposed to TTFields with a frequency of 100 or 200 kHz and either a 1-1 or 1-0 duty cycle.

**Factor**	**Sum. Sq.**	**D.o.F**	**Mean Sq.**	F	**Prob**$>F$
Frequency	270454.9	1	270454.9	49.67	5.62×10^{-12}
Duty Cycle	26916.7	1	26916.7	4.94	0.0266

2. Videos of HeLa Cell Division

The time-lapse microscopy images of live HeLa cells collected from a spinning disk on an inverted microscope were merged with the velocity function in ImageJ to create videos of a single cell undergoing mitosis. The supplementary material files include five such videos: Videos S1 & S2, which correspond to the unexposed (control) HeLa cells; and Videos S3–S5, for the TTFields-exposed HeLa cells (100 kHz with a 1-0 duty cycle — all other settings used during the exposures are the same as those described in the main article). Each video represents approximately 17 h of live-cell imaging, with images acquired every 10 min. Notably, Videos S3–S5 present real-time evidence of the significant mitotic and cytokinetic arrest experienced by HeLa cells exposed to TTFields. Additionally, the intercellular bridge between the two daughter cells remains intact; an unusual effect that is absent in the unexposed cells.
